# Nuclear myosin I regulates cell membrane tension

**DOI:** 10.1038/srep30864

**Published:** 2016-08-02

**Authors:** Tomáš Venit, Alžběta Kalendová, Martin Petr, Rastislav Dzijak, Lukáš Pastorek, Jana Rohožková, Jakub Malohlava, Pavel Hozák

**Affiliations:** 1 Department of Biology of the Cell Nucleus, Institute of Molecular Genetics, AS CR, v.v.i., Videnska 1083, 142 20 Prague, Czech Republic; 2 Faculty of Science, Charles University in Prague, Albertov 6, 128 43 Prague, Czech Republic; 3 Department of Medical Biophysics, Faculty of Medicine and Dentistry, Palacky University in Olomouc, Hnevotinska 3, 775 15 Olomouc, Czech Republic

## Abstract

Plasma membrane tension is an important feature that determines the cell shape and influences processes such as cell motility, spreading, endocytosis and exocytosis. Unconventional class 1 myosins are potent regulators of plasma membrane tension because they physically link the plasma membrane with adjacent cytoskeleton. We identified nuclear myosin 1 (NM1) - a putative nuclear isoform of myosin 1c (Myo1c) - as a new player in the field. Although having specific nuclear functions, NM1 localizes predominantly to the plasma membrane. Deletion of NM1 causes more than a 50% increase in the elasticity of the plasma membrane around the actin cytoskeleton as measured by atomic force microscopy. This higher elasticity of NM1 knock-out cells leads to 25% higher resistance to short-term hypotonic environment and rapid cell swelling. In contrast, overexpression of NM1 in wild type cells leads to an additional 30% reduction of their survival. We have shown that NM1 has a direct functional role in the cytoplasm as a dynamic linker between the cell membrane and the underlying cytoskeleton, regulating the degree of effective plasma membrane tension.

Myosin 1C belongs to the group of class 1 myosins (named A-H), which are monomeric molecules with relatively short tails through which they interact with cargos and other proteins. In humans, it is transcribed from the MYO1C gene, which gives rise to two additional mRNAs differing at the 5′ UTRs and translation start sites. Consequently, three protein isoforms with different N-terminal sequences are generated: the shortest Myo1c isoform C (referred to as Myo1c); the longer Myo1c isoform B, termed the nuclear myosin I (NM1), which has 16 extra amino acids^1^; and the longest Myo1c isoform A (Myo1c-isoA) with 35 amino acids at the N-end^2^.

All vertebrate class 1 myosins share the same features: they bind actin with their head domain and acidic phospholipids with the pleckstrin homology domain (PH), which is localized in their tail part^3^,^4^,^5^. This implicates their physiological functions – linking membranes and membrane-coated vesicles to actin-rich structures, such as cytoskeleton.

Similar to other class 1 myosins, phospholipid interaction tethers Myo1c to the cell periphery^6^, where it facilitates cell adhesion and spreading^7^,^8^,^9^,^10^,^11^,^12^. Myo1c also facilitates trafficking and exocytosis of vesicles rich in various molecules such as GLUT4^13^ or VEGFR^14^. Furthermore, Myo1c serves as a mechanosensor in the hair cells of the inner ear, where it mediates the opening and closure of ion channels upon mechanical stimuli^15^,^16^,^17^. Finally, Nambiar *et al*. showed that overexpression of Myo1c in NIH 3T3 cells leads to increased membrane rigidity, and therefore points to its role in linking the plasma membrane to the cytoskeleton, similarly to other class I myosins^4^.

In contrast, NM1 has always been considered as a nuclear isoform of Myo1c with important functions in DNA transcription^2^,^18^,^19^,^20^, RNA maturation^21^,^22^, and chromatin remodelling^23^. Moreover, in complex with actin, NM1 has been shown to play an important role in the repositioning of a gene locus^24^ or chromosome site upon gene activation^25^ and in relocation of chromosome territories as a reaction to serum starvation^26^. However, NM1 knock-out (KO) mice did not show any obvious phenotype related to the aforementioned nuclear functions, what was explained by the translocation of Myo1c to the nucleus, where it can fully replace NM1^20^,^27^. We therefore asked whether NM1 could functionally replace Myo1c in the cytoplasm of cells.

In this paper we show that NM1 and Myo1c localize predominantly to the cytoplasm and are enriched at the plasma membrane. Upon loss of NM1, cultured mouse fibroblasts exhibit increased resistance to a hypotonic environment, suggesting the role of NM1 in the maintenance of cell membrane tension. This is further supported by atomic force microscopy, which shows that the loss of NM1 leads to a spatial increase in plasma membrane elasticity around the actin cytoskeleton. These findings suggest that NM1 contributes to the cytoskeleton-plasma membrane interaction and has functions typical for other class 1 myosins.

## Results

### NM1 protein is predominantly localized in the cytoplasm

**Figure 1:**
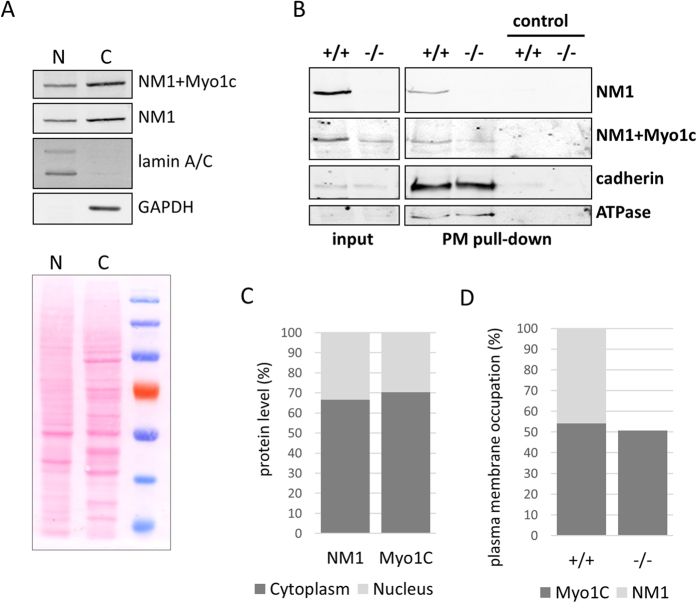
NM1 and Myo1c localize predominantly to the cytoplasm. (**A**) Hela cells were fractionated into the cytoplasm (C) and nuclei (N), and the same amounts of proteins from each fraction were resolved by SDS-PAGE as shown on ponceau stained gel. The purity of each fraction was assessed using lamin A/C and GAPDH as markers. (**B**) Plasma membrane fragments were resolved by SDS-PAGE. ATPase and cadherin were used as markers of the plasma membrane fraction purity and as a loading control. Empty beads were used as negative control. NM1 or both NM1+Myo1c were detected by corresponding antibodies using western blotting. (**C**) Densitometric analysis of the immunoblotting shown in (**A**) shows that there is predominant localization of NM1 and Myo1C in the cytoplasm in comparison to the nucleus. (**D**) Densitometric analysis of the immunoblotting shown in (**B**) suggests that there is the same amount of NM1 as Myo1c at the plasma membrane and that KO of NM1 does not cause translocation of Myo1c to/from the plasma membrane. In both analyses, NM1 and Myo1c are detected by different antibodies; therefore, the signal of these antibodies was normalized to the known amount of purified NM1-Flag present within the same gel (not shown) and used for quantification. The experiment was repeated three times with consistent results.

We have shown previously that NM1 and Myo1c proteins are localized in the cell nucleus^27^ and in the cytoplasm in similar ratios^20^. However, the predominant localization of NM1 in cellular compartments has not been fully described. We therefore prepared nuclear and cytoplasmic fractions from adherent HeLa cells, and compared the amount of NM1 and Myo1c in both compartments by western blotting (Fig. 1A). By using the antibody specific for the N-terminal peptide of NM1 and the antibody against the tail domain (detecting both NM1 and Myo1c), we have shown that both proteins are predominantly localized in the cytoplasm (approximately 70%), as shown by densitometric analysis of the immunoblotting (Fig. 1C.). A similar ratio of NM1 distribution was also observed in lungs tissue^27^.

To measure the amount of NM1 and Myo1c at the plasma membrane, the surface of NM1 wild type (WT) and knock-out (KO) mouse skin fibroblasts was biotinylated and the membrane fragments were isolated using avidin-coupled agarose beads. The plasma membrane fragments were then resolved by SDS-PAGE and proteins detected by western blot (Fig. 1B). Densitometric analysis of the immunoblot suggests that at the plasma membrane the amount of NM1 is the same as Myo1c and the lack of the NM1 in the KO cells does not lead to the translocation of Myo1c to/from the plasma membrane (Fig. 1D).

### Plasma membrane enrichment of NM1 is dependent on the interaction with phosphoinositides

**Figure 2:**
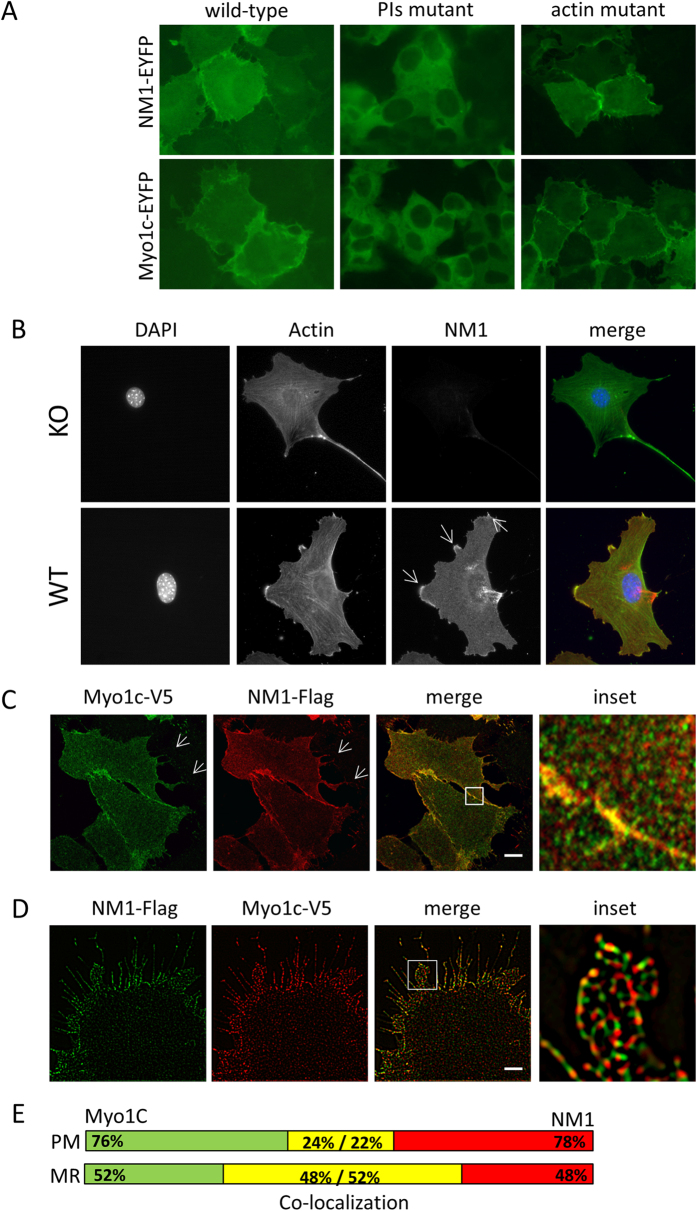
NM1 and Myo1c are enriched at the plasma membrane. (**A**) Wild-type NM1-EYFP or Myo1c-EYFP and their respective mutants (actin-binding or PI-binding) were stably expressed in U2OS cells, and their localization was observed by wide-field fluorescence microscopy. Plasma membrane association is disrupted upon mutation of the PI-binding site of myosins. (**B**) NM1 wild type and KO skin fibroblasts were fixed and immunostained with NM1 and actin antibodies. NM1 is co-localizing with actin in focal adhesions and membrane ruffles. Deletion of NM1 does not cause any alteration in actin cytoskeleton structure. (**C**) NM1-Flag and Myo1c-V5 were co-expressed in the U2OS cells and detected by confocal fluorescence microscopy. Both proteins co-localize at the plasma membrane and in membrane ruffles (arrows). (**D**) Localization of NM1-Flag and Myo1c-V5 was inspected by SIM. Both proteins are localized at the plasma membrane in a mosaic pattern and do not overlap entirely. (**E**) Visualisation of co-localization measurement of Myo1C and NM1 from inset pictures in 3C and 3D. PM – plasma membrane, MR – membrane ruffles. Scale bars, 5 μm (**C**) and 2 μm (**D**).

As mentioned above, the PH domain mediates the interaction of Myo1c with acidic phosphoinositides (PIs), tethering Myo1c to the plasma membrane^6^,^28^. Since the same PH domain is present in NM1, we prepared mutants lacking the PI-binding ability in both NM1 and Myo1c (K908A and K898A, respectively), and observed their localization in human osteosarcoma (U2OS) cells by wide-field fluorescence microscopy. Our results revealed that both, wild-type NM1 and Myo1c localize to the cytoplasm and are enriched at the plasma membrane. However, after mutation in the PI-binding site, both NM1 and Myo1c detach from the plasma membrane and translocate to the cytoplasm. In contrast, mutation in the actin-binding site of either NM1 or Myo1c (RK605AA and RK598AA, respectively) did not affect their association with the plasma membrane (Fig. 2A).

To conclude, NM1 binds to the plasma membrane via the same PH domain as Myo1c in an actin-independent manner.

### NM1 and Myo1c are localized in mostly non-overlapping domains of the plasma membrane

Since both NM1 and Myo1c localize to the plasma membrane, we further investigated whether NM1 occupies the same regions or localizes to distinct areas. Firstly, we observed localization of endogenous NM1 in WT and NM1 knock-out skin fibroblasts by immunofluorescence staining. In KO cells, actin cytoskeleton and overall cell organisation were not affected even though NM1 was completely missing (Fig. 2B). In WT cells, NM1 is localized in the cytoplasm and at the plasma membrane, where it highly co-localizes with actin rich structures, such as focal adhesions or membrane ruffles similarly to Myo1C localization (Fig. 2B, arrows). Myo1c shares the same protein sequence as NM1, therefore there are no specific antibodies recognising just the Myo1C isoform, and we were unable to compare localization of endogenous NM1 and Myo1C together. Therefore, we co-expressed NM1 fused to Flag-tag (NM1-Flag) together with Myo1c fused to V5-tag (Myo1c-V5) in U2OS cells and followed their localization pattern by confocal fluorescence microscopy. We confirmed our previous observations by finding that both NM1-Flag and Myo1c-V5 are enriched at the plasma membrane. Moreover, we observed that the two proteins co-localize, especially at the leading edge and at the membrane ruffles (Fig. 2C, arrows and inset). In other regions, NM1 and Myo1c seem to be present rather next to each other, as shown by Mander’s coefficients showing that only 24% of Myo1c co-localizes with 22% of NM1. Since better resolution can be achieved by super-resolution microscopy, we employed structured illuminated microscopy (SIM). It revealed that even at the membrane ruffles or at the leading edges, NM1 and Myo1c localize next to each other and overlap only partially. However, the overlap is higher in comparison to the plasma membrane – 51% of Myo1c overlap with 48% of NM1 (Fig. 2E). Interestingly, no preferential enrichment of one of the isoforms can be found– they seem to associate with the plasma membrane phosphoinositides (PIs) without showing any spatial preference (Fig. 2D). Taken together, these data suggest similar roles for both isoforms at the plasma membrane as they occupy the same territories.

### Loss of NM1 increases resistance to hypotonic stress

Phenotyping of NM1 knock-out mice has not shown any obvious phenotype related to the plasma membrane function^20^ and NM1 KO cells do not differ in size or spreading (Supplementary Fig. 1). This suggests synergistic and overlapping functions of the class 1 myosins at the plasma membrane. Therefore deletion of one of the myosins does not lead to a phenotype under standard conditions. However, under stressing conditions, such deletion could have a severe effect on the cells.

To test that, we challenged NM1 KO and WT cells with hypotonic stress, during which cells respond to the changes in the osmotic pressure of the surrounding environment by a rapid increase of their volume.

**Figure 3:**
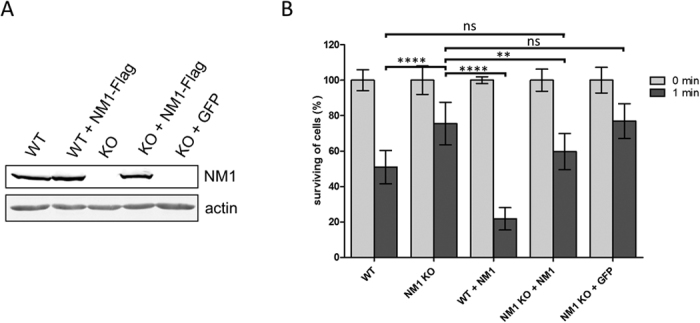
Knock-out of NM1 causes increased resistance to hypotonic conditions. (**A**) WT and NM1 KO mice were used for preparation of mouse skin fibroblasts. By exogenous expression of NM1-Flag in WT and NM1 KO cell lines, WT+NM1 and NM1 KO+NM1, respectively were prepared by lentivirus transduction. NM1 KO+GFP was used as a negative control (**B**). All cell lines were subjected to hypotonic conditions for 1 min followed by 2 hours of recovery under normal growth conditions. NM1 KO fibroblasts show significantly higher ability to resist hypotonic conditions than WT cells. Moreover, WT fibroblasts expressing extra NM1-Flag have lower ability to survive than WT cells. NM1 KO fibroblasts expressing NM1-Flag show similar survival rate as WT, which correlates with the amount of NM1 expressed by both cell lines. The KO-GFP cell line was used as a negative control of the lentivirus transduction effect on hypotonic stress survival. All measurements were done in triplicates. Values are shown as mean ± S.D and statistical significance is presented as ^ns^ p > 0.05, **p < 0.01, ****p < 0.0001, respectively.

We prepared KO and WT fibroblasts stably expressing NM1-Flag or control GFP (Fig. 3A) and subjected them to ddH_2_O for 1 min in parallel with normal WT and KO cells. Surprisingly, NM1 KO fibroblasts exhibited significantly higher ability to survive short time hypotonic stress in comparison to WT cells, which died after plasma membranes bursting. Moreover, when NM1-Flag was expressed in WT fibroblasts, the survival rate was further reduced by an additional 30%, confirming the negative effect of NM1 on the cell survival. In rescue experiments, NM1-Flag exogenous expression in KO cells significantly decreased survival of these cells to almost WT level, while KO cells expressing control GFP kept the same survival rate as KO cells (Fig. 3B).

Taken together, these data suggest that NM1 plays an important role in the maintenance of appropriate volume and osmotic pressure of the cell and possibly regulates plasma membrane tension. This regulation can be direct by linking the plasma membrane to the underlying actin cytoskeleton or indirect by changing the gene expression of some membrane tension regulators or osmosensors.

### NM1 knock-out doesn’t affect expression of proteins regulating plasma membrane tension

To exclude the possibility that NM1 affects membrane elasticity indirectly by changing the gene expression of membrane regulators; and to better understand the pathways in which NM1 is active, we performed microarray analysis from NM1 KO and WT mice. We chose mouse organs with the highest expression of NM1^29^ - lungs and heart, as well as skin fibroblasts which were used in most of the experiments. The analysis revealed 222 statistically significant genes (p < 0.05) with at least 2-fold change in expression (Supplementary Table 1), from which 23 were found in lungs, 102 in heart and 97 in skin fibroblasts. Only known genes were taken into account, all the predicted or non-identified genes were omitted. We performed a gene ontology (GO) enrichment analysis of all genes using PANTHER gene analysis tool^30^,^31^ and also manually verified the results in gene databases Uniprot and NCBI-NLM-NIH.

**Figure 4:**
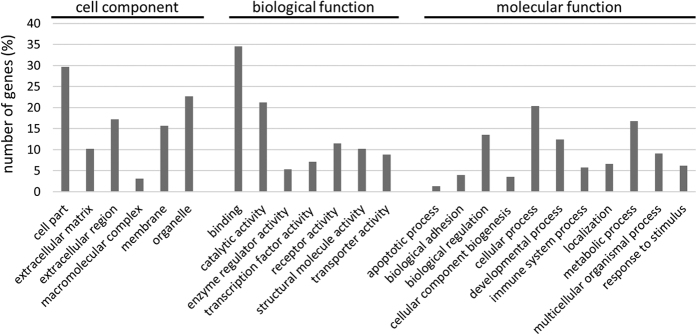
Gene ontology analysis revealed different localization and function of gene products affected by NM1 deletion. NM1 KO and WT cells as well as lung and heart tissues were used for expression profiling. Genes with ≥2 fold up- or down-regulation were classified by gene ontology software to cellular component, biological process and molecular process categories. Number of genes is represented as relative percentage of genes in each category.

Deletion of NM1 protein affects several pathways inside the cell and gene products with changed expression can be found in almost all cellular compartments (Fig. 4). Affected proteins which are located to the nucleus and nucleolus act as transcription factors affecting development, differentiation and proliferation of various types of cells, especially of immune and nervous systems. Many of membrane or transmembrane proteins are receptors on the outer side of plasma membrane and are coupled with G-proteins to trigger downstream signaling. Importantly, there are molecules which mediate cell-cell or cell-matrix adhesion and affect cell motility. In the whole screen we found 12 ion channels-associated proteins that transport various ions or molecules across the plasma membrane. The big portion of proteins is cytoplasmic, playing a role in various processes such as protein processing, signaling events or molecular trafficking. Several proteins are located in either endoplasmic reticulum, Golgi complex or mitochondria. These represent enzymes of lipid metabolism, proton transporters and proteins involved in vesicle trafficking. Proteins that localize in the extracellular space are mostly extracellular matrix components which help to assemble cells into the tissues and to maintain cell functions in connection to surrounding environment.

Interestingly, we have found Myo1C to have changed expression in this screen, which is in contrary with our previous results showing that expression of Myo1C isoform is not changed in NM1 KO cells^20^. This is caused by limitations of the Affymetrix GeneChip Array, which cannot differentiate between NM1 and Myo1C isoforms and contain just general probes for Myo1C. Therefore, deletion of NM1 is recognised in the microarray chip as a decrease of overall Myo1C signal.

Since NM1 deletion leads to increased plasma membrane elasticity and higher survival under hypotonic conditions, we focused on proteins affecting actin cytoskeleton structure, cell adhesion and various ion channels which can function in the pathways triggered after cell swelling and membrane organisation. We found several genes with changed expression in NM1 KO cells in all three categories (Table 1), however none of them were described in processes related to the regulation of membrane tension or cell swelling.

We therefore hypothesize that different membrane tension in NM1 KO cells is most likely not caused by changes in Pol II transcription of some membrane regulators, but rather by direct function of NM1 at the plasma membrane.

### Loss of NM1 leads to higher membrane elasticity

We showed above that NM1 localizes to the plasma membrane, as has been previously demonstrated for other members of class 1 myosin group. Moreover, hypotonic stress response of NM1 KO cells suggests possible NM1 function in regulating plasma membrane tension. However, a more direct approach is needed to provide quantitative evidence supporting the role of NM1 in the regulation of cell membrane dynamics. Therefore, we decided to perform single-cell biomechanical measurements of the cell membrane properties using atomic force microscopy (AFM). We used primary skin fibroblasts derived from NM1 knock-out and wild-type mice (Fig. 3A).

Because of the variable range of cellular morphologies across WT and NM1 KO fibroblasts, we measured the elasticity of cells at the specific regions directly above the nucleus, which was a morphological feature clearly recognizable in all AFM scans.

**Figure 5:**
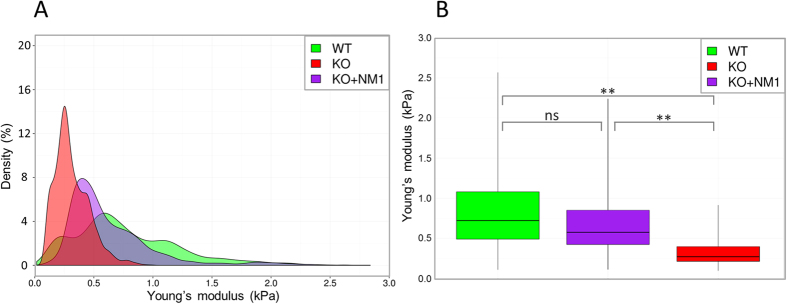
NM1 contributes to the stiffness of the plasma membrane. The elasticity of the plasma membrane of WT, KO and KO expressing NM1-Flag (KO+NM1) cells was measured by AFM. While WT cells display a wide range of elastic properties, NM1 KO cells have a narrow distribution of YM and are generally more elastic. In a rescue experiment, KO+NM1 cells show intermediate elasticities between KO and WT cells. Young’s modulus values are visualised as density plots, where density refers to the relative likelihood for the Young’s modulus to take on a value in the given range (**A**) or as boxplots (**B**).

The overall distribution of Young’s modulus (YM) values of WT and KO cells indicate, that while WT cells show a wide range of elastic phenotypes and, on average, appear to be more rigid (average YM is 0.77 kPa ± 0.38 kPa), NM1 KO cells have consistently higher membrane elasticity (average YM 0.29 ± 0.12 kPa). Importantly, exogenous expression of NM1 in KO cells (KO+NM1) leads to reversion of the phenotype with intermediate values of YM (0.57 kPa ± 0.27 kPa) in these cells (Fig. 5A,B).

To eliminate the possibility that observed results are caused by the underlying nucleus, we measured membrane elasticity over the region closer to the cell periphery, outside of the nuclear area. The calculated mean values of YM for WT, KO and KO+NM1 cells were: 0.94 kPa ± 0.47 kPa, 0.32 kPa ± 0.14 kPa and 0.75 kPa ± 0.4 kPa; respectively (data not shown). This correlates with the previous results measured over the nuclear area.

In conclusion, single-cell biomechanical measurement of cell membrane properties by AFM revealed that cells lacking NM1 have higher membrane elasticity in comparison to WT cells and this phenotype can be reverted by expression of the exogenous NM1 in KO cells.

### NM1 directly influences membrane elasticity by binding to the actin cytoskeleton

**Figure 6:**
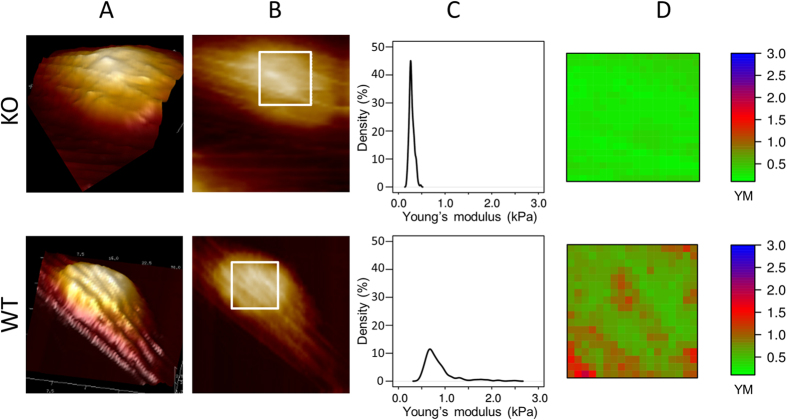
NM1 links the plasma membrane to underlying cytoskeletal structures. (**A**) 3D cell reconstructions of representative cells with different morphological features are shown. Colours represent the relative height values in each of the respective images (black – lowest, white – highest). (**B**) Height maps of representative NM1 KO and WT cells with highlighted membrane regions that were included in the analysis are shown. (**C**) Density plots of YM values calculated from highlighted areas of the cell surface are shown. They are in agreement with previous results, showing a wide distribution of YM values in WT in comparison to the narrow range of YM in KO cells. (**D**) Elasticity maps show striking differences in spatial distributions of YM values measured in NM1 KO and WT cells. The position and shape of actin filaments is reflected in elasticity maps of WT membranes, while in KO cells no such influence from the actin cytoskeleton can be seen. The colour scale shows increasing YM values.

We showed previously by immune-fluorescence staining that actin cytoskeleton is not affected by the deletion of the NM1 protein (Fig. 2B). To test whether the sub-membrane cytoskeleton affects the cell membrane elasticity, we prepared elasticity maps of individual Young’s modulus values for cells with visible actin network (as seen in 3D and height AFM maps of whole cells (Fig. 6A,B)). The density plots of YM values from selected area (Fig. 6B, square) were in agreement with previous results, showing a wider distribution of YM values in WT cells in comparison to the narrow distribution of YM in KO cells (Fig. 6C). However, the elasticity maps showed a striking difference in the spatial distributions of YM values measured in NM1 KO and WT cells. WT cells showed a dramatically higher heterogeneity in the distribution of elastic properties (Fig. 6D), with an intriguing correlation with the underlying actin cytoskeleton visible in 3D and height images (Fig. 6A,B). In WT cells, the position and shape of actin filaments are clearly reflected in elasticity maps of WT membranes, indicating that their presence influenced the apparent membrane stiffness. This was in striking contrast to NM1 KO cells. Their elasticity maps were uniform and more homogeneous, regardless of the presence of visible actin filaments (Fig. 6A) and had a high degree of spatial homogeneity of low elasticity values (Fig. 6D).

Taken together, spatial analysis of elasticity distribution patterns suggests that actin filaments do not have an effect on the plasma membrane elasticity themselves, but more likely, NM1-mediated linkage between actin filaments and the plasma membrane influences membrane elasticity.

## Discussion

Recently, significant progress has been made in the Myo1c field. The studies by Dzijak *et al*. and Ihnatovych *et al*.^2^,^27^ revealed that not only NM1, but all three isoforms encoded by the MYO1C gene enter the nucleus via common NLS. On the top of that, we showed that Myo1c, previously described as an exclusively cytoplasmic isoform, can compensate for the loss of NM1 in the nucleus, and that the two isoforms are interchangeable in the nuclear processes, especially regarding transcription^20^. Therefore, we studied whether NM1 protein, previously described as exclusively nuclear, can also be found in the cytoplasm. We demonstrated that NM1 is tethered to the plasma membrane by interaction with plasma membrane-associated PIs. This finding suggests that NM1 has similar functions in the plasma membrane-associated processes as was described for other class 1 myosins^32^.

Moreover, we found that the levels of NM1 and Myo1c at the plasma membrane are comparable, as previously reported in the nucleus, and that the vast majority of both proteins is localized in the cytoplasm.

However, when we employed super-resolution microscopy, we did not observe complete co-localization of NM1 and Myo1c at the plasma membrane and both proteins seem evenly distributed next to each other. This suggests that they both contribute to the processes related to the plasma membrane, and each of them randomly occupies free PIs integrated in the plasma membrane. The partial co-localization of both myosins can be explained by different distribution of phosphoinositides at the plasma membrane. Structural studies of the crystallised PH domain with PIs have shown that one PH domain is able to bind just one PI at a time^33^. Therefore, the distribution of myosins at the plasma membrane copies the distribution of PIs. Places with higher amounts of PIs also contain higher amounts of both myosins, and their subsequent co-localization is higher. This is in agreement with our data showing that co-localization of both isoforms is higher at the membrane ruffles and at the leading edge, where PIs are also more concentrated^34^.

We have shown that upon deletion of NM1, cells exhibit higher ability to tolerate strong hypotonic conditions. After osmotic cell swelling, numerous pathways are triggered and activate various molecules including ion channels, aquaporins, cell-adhesion molecules, cytoskeletal proteins and molecules remodelling membranes^35^. The aim of all these processes is to restructure the cytoskeleton and the plasma membrane enough to prevent cell bursting. However, under strong hypotonic stress, cells are unable to engage those processes sufficiently on time and their volume increases rapidly. This leads to the rupture of the plasma membrane that is bound to the actin cytoskeleton and to subsequent cell death^36^. Therefore, upon mild hypotonic conditions, myosins have a positive effect on cells, allowing proper exocytosis and membrane rearrangements^7^,^13^,^37^. In contrary, under strong hypotonic conditions, strong linkage between the plasma membrane and the actin cytoskeleton can be deleterious as it interferes with the increasing cell size, which leads to the rupture of the plasma membrane. We hypothesize that a reduction of membrane linkage to the cytoskeleton by deletion of NM1 allows cells to increase their volume without membrane breakage and therefore they can survive short term hypotonic stress.

To support our hypothesis, we measured membrane elasticity of cultured fibroblasts by AFM. We noticed that in WT cells, the stiffness of the membrane correlates with the presence of the underlying actin cytoskeleton. In contrast, in KO cells, no such correlation have been found and the stiffness of the membrane is generally lower, supporting the idea that NM1 regulates plasma membrane tension via its linkage to the underlying actin cytoskeleton. To abolish the possibility that the observed phenotypes are caused by the depletion of Myo1C from the membrane due to a translocation to the cell nucleus to compensate for NM1 loss, we prepared plasma membrane fractions from NM1 KO and WT skin fibroblasts. Upon deletion of NM1, the expression levels of Myo1C does not change^20^ and there is no reduction of Myo1C levels at the plasma membrane, suggesting a direct function of NM1 at the plasma membrane.

Another question is, whether NM1, as an important player in Pol II transcription, can affect membrane tension indirectly by changing the expression of the cytoskeletal or plasma membrane proteins. We therefore performed microarray analysis of NM1 KO and WT cells and tissues, but we did not found any known regulators of plasma membrane tension. This is in agreement with recent paper from Almuzzaini, who performed ChIP-Seq analysis of possible NM1 targets in Pol II mediated transcription^38^. GO analysis of genes found in their study does not show any known proteins which maintain effective plasma membrane tension.

Based on these results, we propose that NM1 creates a dynamic link between the plasma membrane and the cytoskeleton and thus contributes to the maintenance of the membrane tension, as has been already described for other class 1 myosins^32^. We hypothesize that as a consequence of NM1 deletion, the overall number of myosin molecules at the plasma membrane is reduced, the membrane binds to the cytoskeleton less tightly and the cells are more susceptible to swelling. In contrast, WT cells and cells with exogenous overexpression of NM1 have overall higher levels of class 1 myosin molecules at the plasma membrane, and the link between the membrane and the cytoskeleton becomes tighter. This is in agreement with the previous finding that overexpression of other class 1 myosin molecules caused an increase in the rigidity of the plasma membrane^4^. Since NM1 KO cells did not show any difference in size, spreading or motility, we propose that the basic level of membrane tension is sufficiently maintained by other class 1 myosins^4^. After exposure to stress conditions, cells display differences in their mechanical properties. Therefore, NM1 seems to participate in the maintenance of an effective plasma membrane tension by serving as an additional regulatory linker. However, the regulation and dynamics between different myosin 1 members at the plasma membrane are not yet known and need further investigation. For example, the N-terminal extension of NM1 does not affect its membrane localization since the amount of NM1 and Myo1c isoforms at the plasma membrane is the same, but as a part of the motor head domain it could affect the binding of NM1 to the actin cytoskeleton. It is also possible that multiple pathways contribute to the phenotype observed under stress conditions, because NM1 is widely spread all over the plasma membrane, not being exclusively bound to actin filaments and could therefore also act in other processes.

Interestingly, an increasing number of studies have reported that cancer cells have significantly higher elasticity and deformability than control cells derived from healthy tissues^39^,^40^,^41^,^42^,^43^,^44^,^45^,^46^,^47^,^48^. These data resemble our observations of the elastic phenotype of the WT and NM1 KO cells, which show very similar relative patterns of the Young’s modulus value distribution. Therefore, we cannot exclude the possibility that alteration of myosins 1 at the plasma membrane can affect metastatic potential of the cells. But to answer this question properly, additional experimental approaches and new experimental models will have to be applied.

## Materials and Methods

### Ethic statement

All animal experiments and work with human and mice cell lines were carried out in accordance with the approved guidelines. All experimental protocols were approved by the Ministry of Agriculture of the Czech Republic, and the ethic committee of the Institute of Molecular Genetics ASCR (animal experiment license no. 40/2009 and 186/2010).

### Plasmids and cell lines

HeLa and U2OS cell lines were cultured in Dulbecco’s modified Eagle’s medium supplemented with 10% foetal bovine serum. Mouse skin fibroblasts were derived from the WT and KO mice and cultured in Dulbecco’s modified Eagle’s medium supplemented with 10% foetal bovine serum^20^. All cell lines were cultured in a humidified incubator at 37 °C, 5%CO_2_.

NM1 fused to C-terminal EGFP and/or to Flag tag was cloned into pCDH-CMV-MCS-EF1-Neo plasmid using standard molecular biology techniques. Lentivirus, generated using pMD2.G and psPAX2 packaging plasmids, was used for transduction of U2OS and mouse skin fibroblasts. Stable recombinants were selected using G-418.

NM1-Flag (cloned in the pCMV-Tag4B) and Myo1c-V5 (cloned into pCDNA3.1-V5/His) were delivered into U2OS cells by transfection. Lipofectamine 2000 (Life Technologies) was used according to the manufacturer’s protocol.

### Cell fractionation

Cells were fractionated as described previously^49^. Briefly, Hela cells were washed twice with ice-cold PBS, resuspended in ice-cold hypotonic buffer (10 mM Hepes, pH 7.9, 1.5 mM MgCl, 10 mM KCl, 0.5 mM DTT, and protease inhibitors) and incubated on ice for 15 min. Cells were broken in a Dounce homogenizer with a tight pestle and centrifuged at 2300 g for 5 min. The supernatant, which was considered as a cytoplasmic fraction, was mixed with SDS-PAGE loading buffer, sonicated and cleared by centrifugation at 16,000 g. The crude nuclear pellet was further purified over a sucrose cushion: the pellet was resuspended in 0.25 M sucrose, layered over 0.88 M sucrose and centrifuged at 2800 g. The final nuclear pellet was resuspended in the SDS-PAGE loading buffer, sonicated and cleared by centrifugation at 16,000 g. Total protein level was measured by BCA analysis and 20 μg of protein samples were resolved by SDS-PAGE and transferred onto nitrocellulose membrane. As a loading control between nuclear and cytoplasmic fraction was used ponceau staining of SDS-PAGE gel.

### Isolation of plasma membranes

Surface proteins of WT and KO mouse skin fibroblasts were biotinylated using 0.25 mg/ml EZ Sulfo NHS-SS-biotin (Thermo) for 30 min at 4 °C. After that, cells were washed and lysed in 25 mM HEPES pH 8, 500 mM NaCl, 0.5% SDS and 1% Triton X-100 supplemented with protease inhibitors. After sonications, the lysate was cleared by centrifugation and membrane fragments were bound to NeutrAvidin sepharose beads (Thermo). Bound proteins were washed, eluted by boiling in 1xSDS-PAGE loading buffer containing 100 mM DTT. Protein amount was analysed by BCA analysis and 20 μg of protein samples were resolved by SDS-PAGE. Proteins were transferred to nitrocellulose membrane and immunodetected by respective antibodies. ATPase and cadherin were used as markers of the plasma membrane fraction purity and as a loading control.

### Hypotonic stress survival experiments

Cells were seeded at the same densities onto a 24-well plate and kept for 24 hours under normal conditions. Then, the growth medium was discarded, cells were washed with 1xPBS and incubated in ddH_2_O for 1 min. After this period, cells were allowed to recover for 2 hours in normal growth medium under standard conditions. Surviving cells were distinguished by trypan blue exclusion. The experiment was repeated three times in triplicates and the resulting data were statistically evaluated.

### Immunofluorescence and microscopy observation

Cells on coverslips were washed twice with PBS, fixed with 4% paraformaldehyde for 20 min and permeabilised with 0.05% Triton X-100 for 10 min. After 30 min blocking with 1% BSA, cells were incubated for 1 hour with the primary antibody in a wet chamber, washed three times with PBS-T and incubated for 45 min with the secondary antibody. Finally, the cells were washed three times with PBS-T and mounted in Prolong anti-fade reagent (Life Technologies).

Wide-field images were acquired by Leica DM8000. Confocal images were acquired using Leica TCS SP5 AOBS TANDEM. In both cases imaging was performed with 63× immersion oil objective lens, NA 1.4 and a LAS AF software. Superresolution images were acquired using microscope Nikon ECLIPSE Ti-E equipped with an Andor iXon3 897 EMCCD camera and objective CFI SR Apochromat TIRF 100x/1.49 oil. Software NIS-Elements AR 4.20.01 and NIS Elements AR 4.30 were used for capturing and analysis of the images. Resolution obtained at the time of measurements was 120 nm.

### Antibodies

The following antibodies were used for western blotting and immunostaining: anti-NM1 (M3567, Sigma Aldrich); anti-Myo1c (mouse monoclonal^29^); anti-GAPDH (6G5, Acris); anti-V5 (V8137, Sigma Aldrich); anti-lamin A - 133A2, a kind gift from Y. Raymond^50^; anti-actin (A2066, Sigma Aldrich); anti-Flag (M2, Stratagene), anti-Na/K ATPase (Abcam), anti-pan-cadherin (CH-10, Abcam).

### Atomic force microscopy

Measurements of the cell membrane properties were performed in BioScope Catalyst (Bruker) mounted on the stage of a Leica DMI 6000 inverted microscope (Leica) with a heated stage. Silicon nitride DNP-10 probes (Bruker) were mounted on the type B cantilever (Bruker) and the AFM was operated in the PeakForce Tapping mode according to manufacturer’s protocols.

Cells were seeded in 50 mm glass-bottom Petri dishes (WillCo Wells BV) and cultured at standard conditions overnight. Next day, plasma membrane elasticity measurements were performed on the living cells under standard growth conditions. In total, 10 WT, 10 NM1 KO and 7 NM1 KO+NM1 cells were measured and their properties were analysed as described below. Whole cells were fast scanned to obtain overall look on cell shape and height. The analysed scan surface area for measuring of elasticity properties was a 30–40 μm^2^ square and for each cell, a set of 64 × 64 (i.e. 4096) individual force curves were recorded. The following parameters of the AFM system were established during the calibration prior to the measurements: scan rate 0.3 Hz, cantilever deflection sensitivity 35 nm/V, cantilever spring constant 0.197 N/m, tip radius 20 nm, tip half-angle 18^°^, Poisson’s ratio 0.4.

Analysis of the membrane elasticity, was performed using a Bruker NanoScope Analysis application (Bruker, version 1.40) and an open-source R programming environment (version 3.0.1). The values of Young’s modulus (YM) were calculated from each force curve using an Indentation tool of the AFM software by fitting a Sneddon’s model of the tip-sample interaction to the linear portion of the retract force-indentation curve as described previously (51). The final processing and analysis of YM datasets were performed using the R programming environment.

3D and height maps were generated in Bruker NanoScope Analysis application and they are reconstituted from original force curves obtained from whole cells. The height in the picture represents distance of the tip of the probe from the glass bottom at the time of touching the plasma membrane of the cell. The elasticity maps showing the spatial distribution of membrane elasticity were calculated from the corresponding YM dataset exported from the NanoScope Analysis AFM software. Construction of the visual elasticity maps was performed using our own R script, where each point in the map represents the corresponding young’s modulus value.

### Microarray gene-expression profiling and data analysis

Lungs and heart was dissected from WT and KO mice, cells were harvested by trypsinization. Preparation of cDNA, hybridization and gene expression profiling was done by an Affymetrix authorized service provider (AROS Applied Biotechnology A/S, Denmark) using the Affymetrix GeneTitan HT MG-430 PM 24-array plate with the 3′ IVT express labeling kit according to the manufacturer’s protocol. Briefly, following fragmentation, 6.5 μg of cDNA were hybridized for 16 h at 45 °C on the Affymetrix array plate using the Affymetrix GeneTitan system. The array plate was washed, stained and scanned using the Affymetrix GeneTitan system with GCOS 1.4 software. Data analysis was carried out in Genomic Suite Software Partek 6.4 (version 6.09.0602), where the Robust Multichip Analysis was used for background correction. Raw and processed data are stored in open public repository Gene Expression Omnibus (GEO) under accession number GSE75709.

### Statistics

The tests of statistical significance were carried out using an unpaired two-sample Student’s t-test. Values are shown as mean ± S.D and statistical significance is presented as *p < 0.05, **p < 0.01, ***p < 0.001 or ****p < 0.0001, respectively. The plots of the final AFM data are shown as density plots (probability density functions) describing the relative likelihood for the Young’s modulus to take on a value in the given range and as a boxplots, where the height of the boxplot represents an interquartile range, the line horizontally crossing the boxplot represents a median value, and the upper and bottom whiskers extend to the lowest and highest detected extreme values. Mander’s coefficients were calculated using JACoP plugin in ImageJ software^51^.

## Additional Information

**How to cite this article**: Venit, T. *et al*. Nuclear myosin I regulates cell membrane tension. *Sci. Rep*. **6**, 30864; doi: 10.1038/srep30864 (2016).

## Supplementary Material

Supplementary Information

## Figures and Tables

**Table 1:** List of genes related to cytoskeleton, cell adhesion and ion channels with changed expression in NM1 KO cells.

	RefSeq	Fold change	Regulation	Gene symbol	Gene description
**cyto-skeleton**	NM_008659	3.31	down	Myo1c	Unconventional myosin-Ic
	NM_019445	2.64	up	Fmn2	Formin-2
	NM_001284394	2.7	down	Sept4	septin 4
**adhesion**	NM_001040426	2.52	up	Thsd4	Thrombospondin type-1 domain-containing protein 4
	NM_029620	2.59	down	Pcolce2	Procollagen C-endopeptidase enhancer 2
	NM_021362	2.01	up	Pappa	Pappalysin-1
	NM_001033158	2.09	up	Rasl12	Ras-like protein family member 12
	NM_001007221	2.0	up	Adam22	a disintegrin and metallopeptidase domain 22
	NM_000029	2.3	down	Agt	angiotensinogen (serpin peptidase inhibitor, clade A, member 8)
	NM_133654	2.8	down	Cd34	CD34 antigen
	NM_008478	2.4	down	L1cam	L1 cell adhesion molecule
**ion channels**	NM_022004	2.7	up	Fxyd6	FXYD domain-containing ion transport regulator 6
	NM_007390	3.5	up	Chrna7	cholinergic receptor, nicotinic, alpha polypeptide 7
	NM_010595	2.1	up	Kcnab1	potassium voltage-gated channel, shaker-related subfamily, beta member 1
	NM_009200	2.3	up	Slc1a6	solute carrier family 1 (high affinity aspartate/glutamate transporter), member
	NM_001293689	2.7	up	Slc6a17	solute carrier family 6 (neurotransmitter transporter), member 17
