# Supplementary material for: Nuclear myosin I regulates cell membrane tension
Source: Sci Rep. 2016 Aug 2;6:30864. doi: 10.1038/srep30864 (PMC4969604; doi:10.1038/srep30864)
Supplement: Supplementary Information [file srep30864-s1.pdf]

Nuclear myosin I regulates cell membrane tension – supplementary material

Tomáš Venit [a,c]*, Alžběta Kalendová[a]*, Martin Petr[a], Rastislav Dzijak[a], Lukáš Pastorek [a], Jana Rohožková[a], Jakub Malohlava[b] and Pavel Hozák[a]☨

[a] Department of Biology of the Cell Nucleus, Institute of Molecular Genetics, AS CR, v.v.i., Videnska 1083, 142 20 Prague, Czech Republic

[b] Department of Medical Biophysics, Faculty of Medicine and Dentistry, Palacky University in Olomouc, Hnevotinska 3, 775 15 Olomouc, Czech Republic

[c] Faculty of Science, Charles University in Prague, Albertov 6, 128 43 Prague, Czech Republic

* These authors contributed equally to the work

☨ Corresponding author: hozak@img.cas.cz

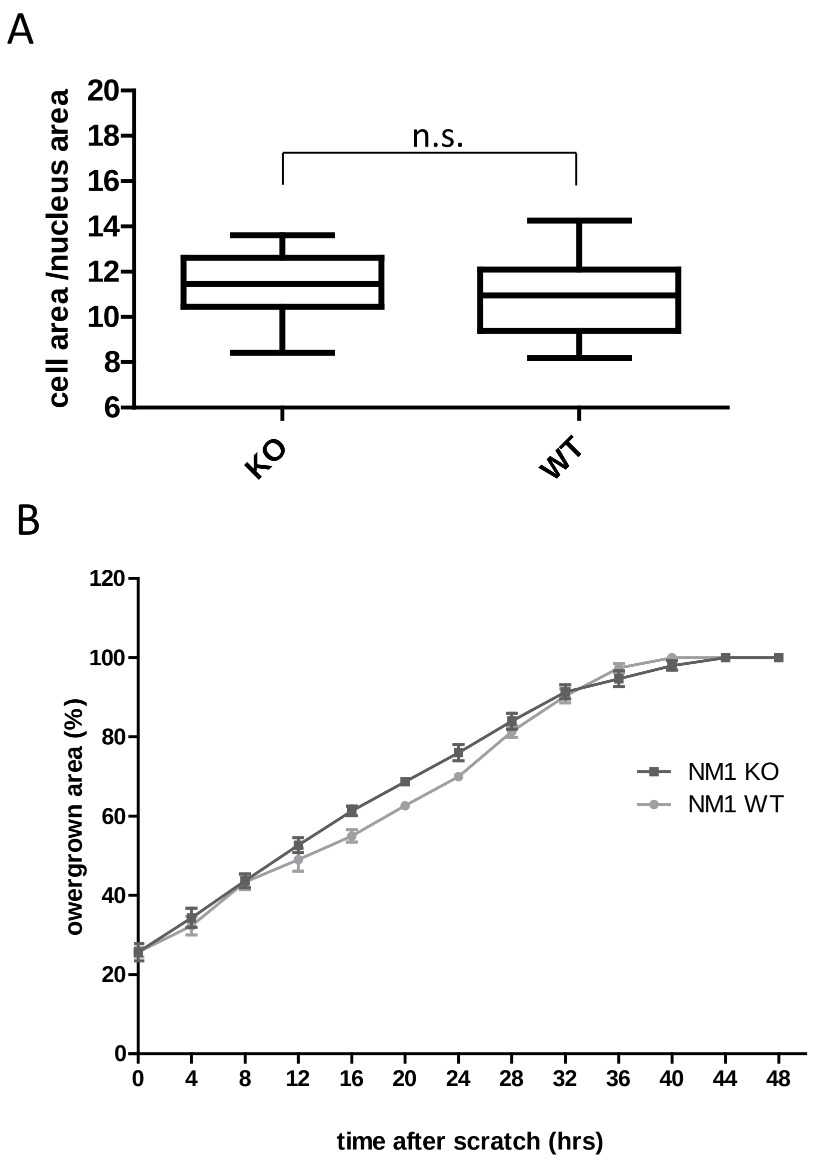

Supplementary figure 1.: NM1 KO does not affect cell size and mobility. A – NM1 KO and WT cells were fixed by 4% PFA and stained by plasma membrane stain and DNA stain DAPI. Size of the nucleus and cell was measured by using ImageJ software. For each cell type, 100 cells were measured. n.s = not significant. **B** – WT and KO cells were grown to 90% confluence, when a scratch by blue tip was performed through the middle of the dish. Subsequently, cells migrating toward the scratch were captured every four hours. Experiment was performed in triplicates and data were analyzed by ImageJ software.

Supplementary Table 1: List of all genes with ≥ 2 fold up- or down-regulation found in microarray analysis from NM1 WT and KO cells and

Fold change	Regulation	p-value	Tissue	Gene symbol	Gene description
2.00	up	0.0016	cells	Mtus2	microtubule associated tumor suppressor candidate 2
2.01	up	0.0256	cells	Adam22	a disintegrin and metallopeptidase domain 22
2.01	up	0.0004	cells	Pappa	pregnancy-associated plasma protein A
2.03	up	0.0012	cells	Trib3	tribbles homolog 3 (Drosophila)
2.03	up	0.0141	cells	Gdnf	glial cell line derived neurotrophic factor
2.03	up	0.0305	cells	Csgalnact1	chondroitin sulfate N-acetylgalactosaminyltransferase 1
2.04	up	0.0206	cells	Tfpi2	tissue factor pathway inhibitor 2
2.04	up	0.0097	cells	Odz4	odd Oz/ten-m homolog 4 (Drosophila)
2.07	up	0.0023	cells	Lmcd1	LIM and cysteine-rich domains 1
2.07	up	0.0000	lungs	W91776	expressed sequence W91776
2.08	up	0.0171	cells	Kcnab1	potassium voltage-gated channel, shaker-related subfamily, beta member 1
2.09	up	0.0170	cells	Rasl12	RAS-like, family 12
2.09	up	0.0037	cells	Stc2	stanniocalcin 2
2.11	up	0.0120	heart	Lrrc27	leucine rich repeat containing 27
2.11	up	0.0019	cells	C1qtnf3	C1q and tumor necrosis factor related protein 3
2.13	up	0.0017	cells	Tlcd2	TLC domain containing 2
2.15	up	0.0000	cells	Id4	Inhibitor of DNA binding 4
2.19	up	0.0000	cells	Dlg2	discs, large homolog 2 (Drosophila)
2.19	up	0.0022	cells	Pitpnm3	PITPNM family member 3
2.20	up	0.0386	heart	Tmprss13	transmembrane protease, serine 13
2.20	up	0.0266	cells	Tnfrsf21	tumor necrosis factor receptor superfamily, member 21
2.20	up	0.0019	cells	Spats2l	spermatogenesis associated, serine-rich 2-like
2.20	up	0.0484	lungs	Ifi27l2a	interferon, alpha-inducible protein 27 like 2A
2.22	up	0.0110	heart	Tmem51	transmembrane protein 51
2.23	up	0.0001	cells	Cpz	carboxypeptidase Z
2.26	up	0.0008	heart	Rtn4rl1	reticulon 4 receptor-like 1
2.27	up	0.0150	cells	Wnt4	wingless-related MMTV integration site 4
2.30	up	0.0000	cells	Slc1a6	solute carrier family 1 (high affinity aspartate/glutamate transporter), member
2.30	up	0.0070	cells	Sox11	SRY-box containing gene 11
2.37	up	0.0082	cells	Sim2	single-minded homolog 2 (Drosophila)
2.39	up	0.0011	cells	Thg1l	tRNA-histidine guanylyltransferase 1-like (S. cerevisiae)
2.40	up	0.0054	cells	Wisp1	WNT1 inducible signaling pathway protein 1
2.43	up	0.0172	cells	Ccbe1	collagen and calcium binding EGF domains 1
2.44	up	0.0041	cells	Foxl2	forkhead box L2
2.48	up	0.0403	cells	Bmpr1b	bone morphogenetic protein receptor, type 1B
2.52	up	0.0007	cells	Thsd4	thrombospondin, type I, domain containing 4
2.52	up	0.0074	cells	Parm1	prostate androgen-regulated mucin-like protein 1
2.63	up	0.0005	cells	Tnfrsf11b	tumor necrosis factor receptor superfamily, member 11b (osteoprotegerin)
2.64	up	0.0025	cells	Fmn2 /// LOC100	formin 2 /// similar to formin-2
2.65	up	0.0296	cells	Bcl11b	B-cell leukemia/lymphoma 11B
2.65	up	0.0007	cells	Cnr1	cannabinoid receptor 1 (brain)
2.65	up	0.0002	cells	Slc6a17	solute carrier family 6 (neurotransmitter transporter), member 17
2.67	up	0.0230	cells	Ch25h	cholesterol 25-hydroxylase
2.71	up	0.0001	cells	Rab3c	RAB3C, member RAS oncogene family
2.71	up	0.0107	cells	Fxyd6	FXYD domain-containing ion transport regulator 6
2.78	up	0.0012	cells	Car2	carbonic anhydrase 2
2.81	up	0.0006	cells	Dmd	dystrophin, muscular dystrophy
2.84	up	0.0204	cells	Tnmd	tenomodulin
2.90	up	0.0096	cells	Crispld2	cysteine-rich secretory protein LCCL domain containing 2
2.92	up	0.0337	cells	Ptprz1	protein tyrosine phosphatase, receptor type Z, polypeptide 1
2.98	up	0.0023	cells	LOC100044883 /	similar to RAB3C, member RAS oncogene family /// RAB3C, member RAS oncogene fami
3.02	up	0.0026	cells	Sema5a	sema domain, seven thrombospondin repeats (type 1 and type 1-like), transmembran
3.02	up	0.0125	lungs	Slfn4	schlafen 4
3.03	up	0.0006	heart	Rbp7	retinol binding protein 7, cellular
3.07	up	0.0115	cells	Pla1a	phospholipase A1 member A
3.10	up	0.0144	cells	Rpl39l	ribosomal protein L39-like
3.29	up	0.0000	heart	Sftpc	surfactant associated protein C
3.33	up	0.0327	lungs	Nrg4	neuregulin 4
3.48	up	0.0052	cells	Mamdc2	MAM domain containing 2
3.54	up	0.0188	cells	Chrna7	cholinergic receptor, nicotinic, alpha polypeptide 7
3.59	up	0.0237	cells	Onecut2	one cut domain, family member 2
4.36	up	0.0153	cells	Cck	cholecystokinin
4.83	up	0.0000	cells	Moxd1	monooxygenase, DBH-like 1
7.00	up	0.0000	cells	Lrrc15	leucine rich repeat containing 15
7.14	up	0.0000	cells	Zic1	zinc finger protein of the cerebellum 1
7.75	up	0.0019	heart	Scgb1a1	secretoglobin, family 1A, member 1 (uteroglobin)
7.95	up	0.0006	lungs	Pisd-ps3	phosphatidylserine decarboxylase, pseudogene 3
8.99	up	0.0000	cells	Chodl	chondrolectin

9.18	up	0.0011	cells	Lgr5	leucine rich repeat containing G protein coupled receptor 5
11.65	up	0.0476	lungs	Ighg	Immunoglobulin heavy chain (gamma polypeptide)
32.83	up	0.0015	heart	Xaf1	XIAP associated factor 1
2.00	down	0.0029	cells	Greb1 /// LOC10(	gene regulated by estrogen in breast cancer protein /// similar to Greb1 protein
2.00	down	0.0002	heart	Prickle2	prickle homolog 2 (Drosophila)
2.00	down	0.0314	lungs	Itga8	integrin alpha 8
2.00	down	0.0127	heart	Nucb2	nucleobindin 2
2.00	down	0.0169	heart	Emilin1	elastin microfibril interfacer 1
2.01	down	0.0181	heart	Ptgis	prostaglandin I2 (prostacyclin) synthase
2.01	down	0.0400	cells	Zdhhc2	zinc finger, DHHC domain containing 2
2.01	down	0.0275	heart	Dcaf12l1	DDB1 and CUL4 associated factor 12-like 1
2.02	down	0.0085	heart	Endod1	endonuclease domain containing 1
2.02	down	0.0080	lungs	Ccl17	chemokine (C-C motif) ligand 17
2.02	down	0.0390	cells	Gprin3	GPRIN family member 3
2.02	down	0.0017	cells	Dpp8	dipeptidylpeptidase 8
2.02	down	0.0451	heart	Col1a2	collagen, type I, alpha 2
2.02	down	0.0016	heart	Mrc2	mannose receptor, C type 2
2.03	down	0.0186	heart	Entpd1	ectonucleoside triphosphate diphosphohydrolase 1
2.03	down	0.0066	heart	Col18a1	collagen, type XVIII, alpha 1
2.03	down	0.0015	heart	Ucp2	uncoupling protein 2 (mitochondrial, proton carrier)
2.03	down	0.0258	heart	Enc1	ectodermal-neural cortex 1
2.03	down	0.0370	heart	Tbc1d1	TBC1 domain family, member 1
2.04	down	0.0459	heart	Sytl2	synaptotagmin-like 2
2.04	down	0.0207	lungs	Kcnk1	potassium channel, subfamily K, member 1
2.05	down	0.0480	heart	Pdlim3	PDZ and LIM domain 3
2.05	down	0.0054	lungs	Zfp39	zinc finger protein 39
2.05	down	0.0057	heart	Fam114a1	family with sequence similarity 114, member A1
2.06	down	0.0163	lungs	Zbtb16	zinc finger and BTB domain containing 16
2.06	down	0.0058	heart	Gcnt2	glucosaminyl (N-acetyl) transferase 2, I-branching enzyme
2.07	down	0.0420	heart	Nr4a1	nuclear receptor subfamily 4, group A, member 1
2.07	down	0.0045	heart	Lass6	LAG1 homolog, ceramide synthase 6
2.08	down	0.0025	heart	Acly	ATP citrate lyase
2.08	down	0.0014	cells	Aldh1a7	aldehyde dehydrogenase family 1, subfamily A7
2.08	down	0.0013	heart	Ampd2	adenosine monophosphate deaminase 2
2.08	down	0.0073	heart	Cd44	CD44 antigen
2.08	down	0.0062	heart	Gan	giant axonal neuropathy
2.08	down	0.0393	heart	Creb3l2	cAMP responsive element binding protein 3-like 2
2.10	down	0.0165	cells	Pdzrn4	PDZ domain containing RING finger 4
2.10	down	0.0066	cells	Smc2	structural maintenance of chromosomes 2
2.10	down	0.0005	heart	Glis2	GLIS family zinc finger 2
2.11	down	0.0212	heart	Ccl7	chemokine (C-C motif) ligand 7
2.11	down	0.0050	cells	Car8	carbonic anhydrase 8
2.11	down	0.0306	heart	Csrp1	cysteine and glycine-rich protein 1
2.12	down	0.0095	cells	Tppp	tubulin polymerization promoting protein
2.13	down	0.0148	cells	Il13ra1	interleukin 13 receptor, alpha 1
2.13	down	0.0126	heart	Pdk3	pyruvate dehydrogenase kinase, isoenzyme 3
2.14	down	0.0060	lungs	Fblim1	filamin binding LIM protein 1
2.14	down	0.0018	heart	Colec11	collectin sub-family member 11
2.14	down	0.0274	heart	Slc38a1	solute carrier family 38, member 1
2.14	down	0.0237	cells	Ganab	alpha glucosidase 2 alpha neutral subunit
2.15	down	0.0123	heart	Kcnj3	potassium inwardly-rectifying channel, subfamily J, member 3
2.15	down	0.0174	heart	Sfrp2	secreted frizzled-related protein 2
2.16	down	0.0047	cells	Pdzd2	PDZ domain containing 2
2.16	down	0.0345	cells	Trdn	triadin
2.16	down	0.0111	heart	Calhm2	calcium homeostasis modulator 2
2.17	down	0.0048	lungs	Edn1	endothelin 1
2.17	down	0.0118	cells	Nid1	nidogen 1
2.18	down	0.0202	cells	Pira2	paired-Ig-like receptor A2
2.18	down	0.0342	heart	Ltbp2	latent transforming growth factor beta binding protein 2
2.18	down	0.0004	heart	Wif1	Wnt inhibitory factor 1
2.19	down	0.0443	cells	Ica1	islet cell autoantigen 1
2.19	down	0.0057	lungs		forkhead box F2
2.19	down	0.0067	lungs	Ccbp2	chemokine binding protein 2
2.20	down	0.0497	heart	Tpm2	tropomyosin 2, beta
2.21	down	0.0019	heart	Vcam1	vascular cell adhesion molecule 1
2.21	down	0.0117	heart	Aff3	AF4/FMR2 family, member 3
2.21	down	0.0004	lungs	Fam46b	family with sequence similarity 46, member B
2.21	down	0.0001	heart	Pde1c	phosphodiesterase 1C
2.22	down	0.0354	cells	Ccl9	chemokine (C-C motif) ligand 9
2.23	down	0.0026	heart	Apcdd1	adenomatosis polyposis coli down-regulated 1
2.23	down	0.0069	heart	Slit3	Slit homolog 3 (Drosophila)
2.23	down	0.0339	cells	AU014678	expressed sequence AU014678
2.24	down	0.0416	cells	Klra18	killer cell lectin-like receptor, subfamily A, member 18
2.25	down	0.0007	heart	Soat1	sterol O-acyltransferase 1

2.26	down	0.0007	heart	Hnmt	histamine N-methyltransferase
2.26	down	0.0156	cells	Tpbpb	trophoblast specific protein beta
2.26	down	0.0052	cells	Tmem100	transmembrane protein 100
2.28	down	0.0327	cells	Ccdc3	coiled-coil domain containing 3
2.29	down	0.0237	heart	Gpc6	glypican 6
2.29	down	0.0319	heart	Gucy1a3	guanylate cyclase 1, soluble, alpha 3
2.30	down	0.0361	heart	Cdr2	cerebellar degeneration-related 2
2.31	down	0.0401	cells	Agt	angiotensinogen (serpin peptidase inhibitor, clade A, member 8)
2.33	down	0.0058	heart	Serpini1	serine (or cysteine) peptidase inhibitor, clade I, member 1
2.34	down	0.0015	heart	Dixdc1	DIX domain containing 1
2.34	down	0.0008	lungs	Hhip	Hedgehog-interacting protein
2.37	down	0.0007	heart	Rbms3	RNA binding motif, single stranded interacting protein
2.37	down	0.0372	heart	Sema3d	sema domain, immunoglobulin domain (Ig), short basic domain, secreted, (semaphor
2.37	down	0.0050	heart	Efhd2	EF hand domain containing 2
2.38	down	0.0168	heart	Vasn	vasorin
2.39	down	0.0150	heart	Slc4a1	solute carrier family 4 (anion exchanger), member 1
2.40	down	0.0112	cells	L1cam	L1 cell adhesion molecule
2.41	down	0.0025	heart	Isg20	interferon-stimulated protein
2.44	down	0.0036	heart	Tmem119	transmembrane protein 119
2.44	down	0.0261	cells	Sfi1	Sfi1 homolog, spindle assembly associated (yeast)
2.46	down	0.0478	heart	Tagln	transgelin
2.46	down	0.0073	heart	Foxc2	forkhead box C2
2.47	down	0.0041	heart	Prelp	proline arginine-rich end leucine-rich repeat
2.50	down	0.0139	heart	Stbd1	starch binding domain 1
2.50	down	0.0072	heart	Comp	cartilage oligomeric matrix protein
2.51	down	0.0000	cells	Barx1	BarH-like homeobox 1
2.52	down	0.0024	heart	Cdh11	cadherin 11
2.54	down	0.0117	heart	Aebp1	AE binding protein 1
2.59	down	0.0134	cells	Pcolce2	procollagen C-endopeptidase enhancer 2
2.60	down	0.0281	heart	Myh11	myosin, heavy polypeptide 11, smooth muscle
2.61	down	0.0174	heart	Rgs7	regulator of G protein signaling 7
2.61	down	0.0284	cells	Ddx54	DEAD (Asp-Glu-Ala-Asp) box polypeptide 54
2.61	down	0.0330	cells	Prl2c2 /// Prl2c3	prolactin family 2, subfamily c, member 2 /// prolactin family 2, subfamily c, m
2.62	down	0.0325	heart	Cacna1h	calcium channel, voltage-dependent, T type, alpha 1H subunit
2.62	down	0.0112	heart	Vcan	versican
2.64	down	0.0075	heart	Abi3bp	ABI gene family, member 3 (NESH) binding protein
2.70	down	0.0008	cells	Sept4	septin 4
2.75	down	0.0008	heart	Slc25a37	solute carrier family 25, member 37
2.76	down	0.0458	heart		integrin alpha 8
2.77	down	0.0175	cells	Cd34	CD34 antigen
2.78	down	0.0420	heart	Peg3	paternally expressed 3
2.81	down	0.0001	cells	Il1rn	interleukin 1 receptor antagonist
2.81	down	0.0240	heart	Prss23	protease, serine, 23
2.86	down	0.0045	heart	Fn1	fibronectin 1
2.86	down	0.0003	cells	Hist3h2a	histone cluster 3, H2a
2.86	down	0.0038	cells	Meox2	mesenchyme homeobox 2
2.87	down	0.0004	cells	Nde1	nuclear distribution gene E-like homolog 1 (A. nidulans)
2.88	down	0.0397	heart	Itih5	inter-alpha (globulin) inhibitor H5
2.89	down	0.0155	heart	Kcna1	potassium voltage-gated channel, shaker-related subfamily, member 1
3.02	down	0.0255	heart	Myl9	myosin, light polypeptide 9, regulatory
3.06	down	0.0139	heart	Sulf1	sulfatase 1
3.06	down	0.0108	heart	Slc5a3	solute carrier family 5 (inositol transporters), member 3
3.08	down	0.0310	lungs	Trim16	tripartite motif-containing 16
3.19	down	0.0442	heart	Cd24a	CD24a antigen
3.23	down	0.0066	heart	Tspan2	tetraspanin 2
3.31	down	0.0062	cells	Myo1C	myosin IC
3.37	down	0.0082	heart	Vit	vitrin
3.55	down	0.0164	heart	Fmod	fibromodulin
3.73	down	0.0286	cells	Pfdn5	prefoldin 5
3.81	down	0.0071	heart	Plekha7	pleckstrin homology domain containing, family A member 7
3.95	down	0.0221	cells	Stmn2	stathmin-like 2
4.03	down	0.0033	cells	H2-Aa	histocompatibility 2, class II antigen A, alpha
4.26	down	0.0002	heart	Fam107a	family with sequence similarity 107, member A
4.40	down	0.0315	heart	Alas2	aminolevulinic acid synthase 2, erythroid
4.48	down	0.0239	heart	Mlana	melan-A
5.53	down	0.0015	heart	Fam46c	family with sequence similarity 46, member C
5.67	down	0.0113	heart	Snca	synuclein, alpha
6.28	down	0.0244	heart	Dkk3	dickkopf homolog 3 (Xenopus laevis)
7.25	down	0.0003	heart	LOC640441 /// T	similar to thrombospondin 1 /// thrombospondin 1
7.67	down	0.0269	cells	Bst1	bone marrow stromal cell antigen 1
9.46	down	0.0000	heart	Rab6b	RAB6B, member RAS oncogene family
